# Summary of the primer on tumor immunology and the biological therapy of cancer

**DOI:** 10.1186/1479-5876-7-11

**Published:** 2009-01-28

**Authors:** Yufeng Li, Shujuan Liu, Kim Margolin, Patrick Hwu

**Affiliations:** 1 Department of Melanoma Medical Oncology, the University of Texas, M. D. Anderson Cancer Center, Houston, TX, USA; 2 Department of Medicine Division of Oncology, University of Washington, Seattle, WA, USA

## Abstract

The International Society for Biological Therapy of Cancer (iSBTc) is one of the "premier destinations for interaction and innovation in the cancer biologics community". It provides a primer course each year during the annual meeting to address the most important areas of tumor immunology and immunotherapy. The course has been given by prominent investigators in the area of interest, covering the core principles of cancer immunology and immunotherapy. The target audience for this program includes investigators from academic, regulatory, and biopharmaceutical venues. The program goal is to enable the attendees to learn the current status and the most recent advances in biologic therapies, and to leverage this knowledge towards the improvement of cancer therapy. The 2008 immunologic primer course was held on October 30 at the 23rd Annual meeting of iSBTc in San Diego, CA. Nine internationally renowned investigators gave excellent presentations on different topics. The topics covered in this primer included: (1) cytokines in cancer immunology; (2) anti-angiogenic therapy; (3) end stage: immune killing of tumors; (4) blocking T cell checkpoints; (5) approach to identification and therapeutic exploitation of tumor antigens; (6) T regulatory cells; (7) adoptive T cell therapy; (8) immune monitoring of cancer immunotherapy; and (9) immune adjuvants. We summarized the topics in this primer for public education. The related topic slides and schedule can be accessed online .

## Cytokines in cancer immunology

The development of anti-cancer cytokines is an active area for investigators in the field of cancer immunotherapy. Dr. Mario Sznol, MD (Yale University School of Medicine) gave a comprehensive topic on the application of cytokines in cancer immunotherapy. Both immune or non-immune cells can be the focus of biological rationals for cytokine therapy, including: 1) T cells: to enhance the development, proliferation and/or function of either endogenous or adoptively transferred effector T cells; 2) NK cells: to enhance NK activity and improve ADCC; 3) tumor cells: to upregulate Ag and MHC expression, or induce an anti-proliferative effect; 4) DC/APC: to generate and mature DC/APC *in vitro*, and to increase DC/APC number and function *in vivo*.

Although over 20 cytokines have been developed for the treatment of cancer, only IL-2, IFN-α and TNF-α have been approved in the US and/or Europe for immunologic anti-cancer therapy. Multiple issues for clinical development of cytokines have been highlighted over decades of studies, such as their context-dependent biological effects, secondary effects, and differences in response between individuals. IL-2 was one of the first cytokines to be applied to cancer therapy. IL-2 induces T cell activation and proliferation and stimulates NK cell cytotoxicity; however, IL-2 also causes vascular leak syndrome, which can lead to significant side effects. IL-2 regimens have been tested in several types of cancers, with a 15% response rate only in human metastatic renal cell carcinoma and melanoma. Adoptive cell transfer of tumor infiltrating lymphocytes to lymphodepleted patients with melanoma in combination with high dose IL-2 has been shown to achieve clinical responses in the range of 50%. However, minimal activity of IL-2 in the treatment of other cancers has been observed. Mechanistic studies involving T cells activation, T regulatory cells and B7 co-stimulatory family members are under investigation to address how IL-2 works or fails in therapy. IL-2, IL-15 and IL-21 all belong to the common gamma chain receptor family. Targeting NK, NKT and memory CD8^+ ^T cells, IL-15 exerts its functions preferentially through trans-presentation. Murine models demonstrated that IL-15 enhances *in vivo *anti-tumor activity of adoptively transferred T cells, which is further enhanced in combination with an anti-IL-2 antibody. IL-21 may be a promising candidate for cancer immunotherapy as it has pleiotropic roles in immune cells, yet does not support Treg function. A combination of IL-15 and IL-21 may be a choice for future therapeutic regimens, as suggested by some mouse studies. The clinical experience with IL-12 was also summarized; local administration is recommended due to its excessive systemic toxicity. Other cytokines, such as IL-6, IL-7, Th17, and TGF-β were also discussed in this lecture. Future applications of new cytokines include *in vitro *expansion of antigen-specific T cells and the support for adoptively transferred cells; local application as a vaccine adjuvant; antibodies to neutralize selected cytokines to enhance immune responses; or combination uses, such as with immune modulating monoclonal antibodies (such as anti-CTLA4).

## Anti-angiogenic therapy

Dr. David Cheresh (University of California, San Diego) updated studies on targeting tumor angiogenesis by blocking the VEGF/VEGFR pathway. Growth factors of the VEGF and PDGF families function primarily in a paracrine manner to promote angiogenesis (the sprouting of new blood vessels from pre-existing ones) and vasculogenesis (the generation of new blood vessels where no blood vessels previously exist). Both angiogenesis and vasculogenesis play roles in the formation and maintenance of tumor vasculature and the progression of cancer. VEGF and PDGF bind their corresponding receptors to trigger receptor autophosphorylation and the initiation of downstream signaling processes. The ligation of VEGFR-2 by the majority of VEGF isoforms triggers the proliferation, migration and survival of endothelial cells, which in tumors form the framework of immature new neoplastic vessels. The PDGFs play a role in the regulation of cell proliferation, and function as growth signals for pericytes and Vessel-Smooth-Muscle-Cells (VSMC) that line and stabilize the nascent vessels formed by endothelial cells.

VEGF, through its receptor, attenuates PDGF-mediated pericyte/VSMC coverage of blood vessels. The VEGF receptor interacts with the PDGF receptor to inhibit PDGF signaling. VEGFR-2 pathway blockade (Avastin) increases pericyte coverage and normalizes tumor vessels. Besides vascular cells and tumor cells, myeloid cells can also produce VEGF. In a myeloid-specific VEGF knock out mouse, pericytic coverage was improved. Furthermore, Avastin treatment achieved better tumor control in myeloid-specific VEGF knock out mice compared to wild-type mice. Together, the data provides a mechanism to explain how VEGF/VEGFR blockade increases pericyte coverage, and also challenges us to utilize these agents to effectively treat tumor.

## End stage: immune killing of tumors

The ultimate goal of cancer immunotherapy is to lyse tumor cells with immune mechanisms. Dr. William Murphy (U.C. Davis School of Medicine) described the pathways towards immune-mediated tumor lysis. The basic steps for immune effector cells to kill tumors include target recognition and conjugate formation, followed by tumor lysis or growth arrest. Immune effector elements, including T cells, NK cells, monocyte/macrophages, and antibodies can directly kill tumors through lytic/cytostatic mechanisms by secreting perforin/granzymes, or inducing tumor cytostatis or apoptosis; or indirectly mediate tumor inhibition via attacking tumor supportive elements such as endothelial or stromal cells. Tumor cells escape immune killing by blunting the basic requirements of immune effector cell function and inducing an immuno-suppressive environment. Thus, means to improve target recognition and conjugation, enhance lysis potential, and overcome tumor evasion, will lead to effective tumor killing. Based on the principles of immune killing of tumors, strategies to augment anti-tumor immunity are under investigation or already used for the treatment of cancer, such as cytokine therapy to activate effector cells (Interferon, IL-2, etc), chemoimmunotherapy (Doxorubicin), molecular targeting (proteasome inhibition, HDAC inhibitors), blocking anti-apoptotic machinery (antisense to bcl-2), blocking immune suppression by tumor (COX2 inhibitors, blockade of TGF-β), augmenting effector cell capacities (genetically engineered immune cells that survive and function better in immunosuppressive environments). Dr. Murphy also discussed the measurement of tumor killing. As demonstrated, Bortezomib can sensitize tumor cells to death by inhibiting NF-κB, reducing c-FLIP and stabilizing p53. Bortezomib also enhances the killing through NK cells, as was supported by *in vitro *and *in vivo *long term tumorigenesis assays. The design of assays to reflect and validate *in vivo *tumor killing mechanisms is challenging. The *in vitro *assay may be used for the initial screen, and multiple tumor cells, doses and mechanisms of action with long-term assays should be tested for better evaluation of killing efficacy potential. For *in vivo *models, spontaneous tumors or slower growing orthotopic tumors were suggested in order to mimic the natural tumor microenvironment.

## Blocking T cell checkpoints

The T cell response requires two signals: the first signal is the recognition and binding of the T cell receptor (TCR) to antigen bound within the major histocompatibility complex (MHC) presented by APCs; the second is the binding of costimulatory ligands, expressed on APC, to receptors on the T cells. The discovery of multiple costimulatory molecules that influence the course of T cell activation has increased our appreciation of the complexity of the T cell response. CD28 and cytotoxic T lymphocyte antigen 4 (CTLA-4) are the critical costimulatory receptors that determine the early outcome of stimulation through TCR. CTLA-4 plays a critical role in the down-regulation of T cell responses. Its inhibition may restrict T cell activation during both the initiation and progression of the antitumor response. Thus, blockade of CTLA-4 inhibitory signals during T cell-APC interactions can result in enhanced tumor immunity. Dr. Jim Allison (Memorial Sloan Kettering) reviewed the studies on the anti-CTLA-4 monoclonal Ab to negate this "brake" function. He first presented work using anti-CTLA-4 Ab alone or in combination with other modalities to treat murine tumors. Activation of vasculature in tumors, extravasation and proliferation of T cells, and increased ratios of Teff/Treg and IFN-γ/IL-10 were discovered to be the mechanisms of anti-tumor effects of CTLA-4 blockade in mouse models. It was shown that Teff cells are the major population accountable for the anti-tumor effects of anti-CTLA-4; CTLA-4 blockade in Tregs alone does not significantly contribute to tumor control; while blocking CTLA-4 in both populations is necessary for an optimal anti-tumor response. He then reviewed the studies of lpilimumab, a human CTLA-4 monoclonal Ab, utilized in clinical trials. More than 3700 patients were treated with lpilimumab; clinical responses have been seen in melanoma, renal, prostate, ovarian and Hodgkins lymphoma. 15–20% of response can be seen in melanoma as monotherapy, and this seems to be increased when combined with vaccines. The adverse effects of lpilimumab are manageable with monthly administration, and can be alleviated by spacing out treatments. The critical questions for further clinical development of anti-CTLA-4 to be answered are: the mechanisms involved in the anti-tumor effects; how to distinguish responders from non-responders; the best combinations with conventional therapies or vaccines. Dr. Allison also updated data of other targets for checkpoint blockade and possible candidates for cancer immunotherapy, such as PD-1, B7-H3 and B7x. In summary, the data indicates that checkpoint blockade is a potential strategy to unleash the immune system to maximize T cell responses to multiple targets for cancer immunotherapy.

## Approach to identification and therapeutic exploitation of tumor antigens

Dr. Walter Urba (Earle A. Chiles Research Institute) reviewed the approaches to identify and therapeutically utilize tumor antigens. Tumor antigens can elicit immune responses, which lead to tumor elimination. In most cases in cancer, tumor cells transform and mutate frequently, resulting in immune equilibrium and finally escape immune surveillance. A rational way of fighting cancer is to identify tumor antigens and utilize them in vaccines to boost anti-tumor immunity. Many approaches have been used to discover tumor antigens, including: 1. direct immune approach, starting with T-cells or antibodies that recognize tumors and identifying the antigens by cDNA cloning techniques; 2. reverse immune approaches, starting with candidate antigens that are over-expressed by tumors and determining whether T-cells can recognize these antigens. Numerous human tumor antigens have been discovered using the above approaches, covering shared tumor-specific antigens (MAGE, NY-ESO-1, etc), antigens resulting from mutations (MUM-1, CDK4, etc.), differentiation antigens (MART-1, gp100), overexpressed antigens (p53, HER2/neu), and viral antigens (EBV, HPV16). Ideally, a tumor antigen should be specific and immunogenic, with multiple epitopes and high levels of expression. Ideally, the antigen should be critical for oncogenicity. Finally, the tumor antigen has to be clinically proven to be efficacious in vaccine trials. For example, the cancer/testis antigens (CT Ag) are a group of prominent Ags, such as NY-ESO-1, MAGE, whose expression is restricted in tumors, testis and/or placenta, but not in more than two non-germline normal tissues; CT antigens are immunogenic in cancer patients; their expression may be associated with tumor progression and with tumors of high metastatic potential. Active immunization of cancer patients targeting tumor antigens can be conducted using different strategies, such as antigenic peptides, whole proteins or virus-like particles; recombinant viruses/bacteria/DNA encoding tumor Ag genes; or cells expressing tumor Ags. So far, tumor Ag vaccination in clinical trials has had disappointing results. Several issues have been highlighted, such as loss of Ag expression or MHC on tumor cells post treatment, and lack of sufficient immune adjuvants or trafficking of T-cells to the tumor. However, better antigen selection and methods to overcome tumor escape should improve active cancer immunotherapy in the future.

## T regulatory cells

The scientist who first described T regulatory cells (Treg), Dr. Shimon Sakaguchi (Kyoto University, Japan), updated Treg research in relation to the immunotherapy of cancer. Ever since classical T regulatory cells were discovered utilizing CD4^+ ^CD25^+ ^T cell depletion experiments, tumor immunity has been closely examined in regard to Tregs. Induction of anti-tumor immunity by CD4^+ ^CD25^+ ^Treg depletion was first proved in mouse models. Anti-IL-2 treatment reduced CD25^+ ^Treg, and mice developed autoimmune disease. IL-2 is crucial for self-tolerance maintenance. Foxp3 is a master transcription factor in Tregs, and Foxp3^+ ^Treg have constitutive expression of CTLA-4. CTLA-4 blockade abrogates Treg suppression. Further effective tumor immunity was provoked in Treg-restricted-CTLA-4^-/- ^mice. Through microarray analysis, folate receptor 4 (FR4) was discovered to have high expression on activated Treg cells. Functional analysis indicated that FR4 differentiate activated Teff into Treg, and its blockade leads to Treg depletion *in vivo*, in turn improving tumor rejection. GITR is another molecule preferentially expressed by Treg. DTA-1, an antibody for GITR, can abrogate Treg suppression while not depleting Treg, can reverse Teff/Treg ratio and increase CD4 T cell infiltration into tumors, and can synergize with CTLA-4 blockade to enhance anti-tumor immunity. In summary, several molecules associated with Treg function and maintenance can be targeted for cancer immunotherapy.

## Adoptive T cell therapy

Dr. Philip Greenberg (Fred Hutchinson Cancer Research Center & University of Washington) discussed three major obstacles of adoptive cell therapy and strategies to overcome them for better cancer immunotherapy. First, select optimal tumor antigens for targeting. Active immunization of characterized Ags has been explored for many years and success remains limited. Adoptive cell therapy is an alternative way to isolate and expand antigen specific T cells for potent tumor immunity for the treatment of cancer. Although infused T cells infiltrate tumors and exhibit tumor control in some patients, tumor antigen evasion still remains a major problem. Thus, targeted antigen selection is important for treatment. The solution is to select over-expressed oncogenes indispensable for the tumor phenotype. An effective isolation strategy by enrichment of CD137^+ ^reactive T cells is especially helpful for identifying rare responding T cells. As an example, a novel WT1 epitope restricted by a class I allele was discovered in >40% of leukemia patients. A phase-I clinical trial with WT1 specific T cells has demonstrated T cell persistence and reduced tumor burden in some patients. Second, it is difficult to generate large numbers of high avidity tumor-reactive CD8^+ ^T cells in individual patients in time and maintain their survival *in vivo*. The solution is gene therapy, by engineering T cells with high avidity through insertion of cloned TCRs of known specificity and affinity. T cell avidity can be further improved by mutating low affinity TCRs prior to insertion into host T cells. To improve the survival of transferred T cells *in vivo*, pro-survival molecules/signals or receptor genes are engineered into T cells that inherently survive better *in vivo*. A novel strategy to improve T cell recognition of poorly processed/presented tumor antigens or MHC class I loss tumors, is to create chimeric receptors that take advantage of Ab-recognition structures, which have higher affinities than TCRs and don't require MHC. Chimeric TCR structures can be further modified with costimulatory and/or signal transducing molecules to improve signaling and promote survival. The third obstacle is how to maintain effective T cell response in the hostile micro- and macro-environment created by a progressive tumor. A dual TCR model has been established to address this question. The results show that *in vivo *stimulation of T cells with dual TCR via the non-tolerized TCR can transiently rescue the anti-tumor activity mediated through the tumor-reactive TCR. Finally, molecular disruption of T cell regulatory checkpoints would help transferred T cells resist the tumor inhibitory microenvironment. For example, Cbl-b can be knocked down by siRNA, thus allowing better T cell activation and effective anti-tumor activity. CTLA-4 blockade is another potential strategy to be combined with adoptive cell transfer for effective host responses against tumor.

## Immune monitoring of cancer immunotherapy

Dr. Michael Kalos (City of Hope) emphasized the importance of correlative studies and approaches to achieve comprehensive immune monitoring. Correlative studies are a primary mechanism through which meaningful insight about clinical trials can be obtained. How we perform correlative studies is critical for effective evaluation of years of effort and cost, and patient time and commitment. It is critical to design correlative studies that are as broadly comprehensive as possible, and ensure specimens are appropriately processed and archived for future evaluation. Validation and quality are principles of scientific soundness for correlative assays. Assays should provide meaningful data under specific conditions (qualification), and be established to assure it is working properly and consistently (validation). For translational research, the ability to perform efficient and rational clinical trials is critical for the development of ultimately successful treatments. For cancer immunotherapy, multiple parameters (phenotype and/or function) should be measured simultaneously for comprehensive correlative studies. Several platforms have been developed for performing these studies. For example, at the single cell level, multi-parameter flow cytometry can perform immunophenotyping (subsets, cell status, spectratyping), as well as effector assays (cytolysis, degranulation, proliferation and cytokine production); at the population based level, Q-RT-PCR is broadly used for gene expression assays, and luminex assays can measure not only dozens of cytokines, chemokines, but also the phosphorylation levels of proteins. In summary, correlative studies are critical to guide the development of effective therapies. Studies need to be designed as comprehensively as possible, and to be performed to the highest possible scientific standards to achieve the goal. There is "significant rational and justification" for the support of a qualified facility to perform correlative studies.

## Immune adjuvants

Dr. Karolina Palucka (Baylor Institute for Immunology Research) discussed the natural immune adjuvant, dendritic cells, to help tumor antigen presentation. Multiple signals can mature DC, such as microbial products, tissue damage, and innate/adaptive immune components. DC can be induced into mature status either as tolerogenic (by β-catenin, NO, IL-10) or immunogenic (by type I IFN, IL-12). Great attention has to be paid on the selection of DC as immune adjuvants for vaccination, because different types of DCs have distinct functions, such as pDC, mDC (langerhans DC, interstitial DC). As a perfect example, skin DC can be CD14^+^, DC-SIGN^+ ^(IntDC), or CD1a^+^, Langerin^+ ^(LC-DC). LC-DC are more efficient in CD8^+ ^T cell priming and proliferation than IntDC, thus, LC-DC are better for cross priming/presentation. However, IntDC prime follicular CD4^+ ^T cells more efficiently to induce B cell antibody responses. To design tumor vaccines, peptides (tumor associated Ags) or killed allogenic cancer cells were pulsed onto DCs. Different protocols of DC generation and maturation have been utilized, including CD34-DC pulsed with KLH and GM-CSF and IL-4 generated monocyte derived DC matured with LPS. Cytoxan, which eliminates Treg and reduces IL-10 production, has also been tested in combination with DC vaccines. The future of optimized DC vaccine strategies will be to optimize CTL induction while selecting the proper methods to load DCs *in vitro *or *in vivo *with antigens and simultaneously blocking immunosuppressive elements.

## Summary

In summary, this primer covered many conceptual and practical challenges to understand tumor immunology and leverage this knowledge towards improving the biological therapy of cancer. The expected outcomes after the completion of this program were to enable the participants to 1. discuss immunology as it applies to cancer etiology, biology and therapy; 2. review cellular immunology and host-tumor-immune system interactions, 3. present in depth concepts of humorally-based immune therapies; 4. assess cytokine biology and the role of cytokines in cancer therapy; and 5. evaluate the foundation and methods for clinical trials of biologic/immunologic therapies.

## Authors' contributions

YL and SL drafted the summary, and contributed equally. PH and KM planned, organized and chaired the primer of tumor immunology for the 2008 iSBTC annual meeting, and initiated the idea of summarizing this event. PH critically read, edited and finalized the manuscript. All authors read and approved the final manuscript.

